# The Use of Positron-Emission Tomography–Magnetic Resonance Imaging to Improve the Local Staging of Disease in Myxofibrosarcoma: A Feasibility Study

**DOI:** 10.3390/diagnostics15081039

**Published:** 2025-04-19

**Authors:** Corey D. Chan, Marcus J. Brookes, Tamir Ali, Elizabeth Howell, Petra Dildey, Michael Firbank, Rachel Pearson, Philip Sloan, Simon Lowes, Raj Sinha, John Tuckett, Maniram Ragbir, Thomas Beckingsale, Geoff Hide, Craig Gerrand, Kenneth S. Rankin, George S. Petrides

**Affiliations:** 1 Translational and Clinical Research Institute, Newcastle University Centre for Cancer, Newcastle upon Tyne NE1 7RU, UKkenneth.rankin1@nhs.net (K.S.R.); 2 The North of England Bone and Soft Tissue Tumour Service, Freeman Hospital, Newcastle upon Tyne Hospitals NHS Foundation Trust, Newcastle upon Tyne NE7 7DN, UK; 3 Radiology and Nuclear Medicine Department, Newcastle upon Tyne Hospitals NHS Foundation Trust, Newcastle upon Tyne NE7 7DN, UK; 4 Nuclear Medicine Department, North Cumbria Integrated Care NHS Foundation Trust, Carlisle CA2 7HY, UK; 5 Pathology Department, Newcastle upon Tyne Hospitals NHS Foundation Trust, Newcastle upon Tyne NE7 7DN, UK; 6 Northern Centre for Cancer Care, Newcastle upon Tyne Hospitals NHS Foundation Trust, Newcastle upon Tyne NE7 7DN, UK; 7 Gateshead Health NHS Foundation Trust, Gateshead NE9 6SX, UK; 8 The Bone and Soft Tissue Tumour Service, Royal National Orthopaedic Hospital NHS Trust, Stanmore HA7 4LP, UK

**Keywords:** sarcoma, PET, MRI, imaging, myxofibrosarcoma, tumour, surgery

## Abstract

**Background/Objectives**: Myxofibrosarcomas (MFSs) are aggressive soft-tissue sarcomas (STSs) that often arise in the upper and lower limbs. MFSs are a highly infiltrative sarcoma subtype with a high positive margin rate and poor clinical outcomes. Their management involves multidisciplinary team (MDT) input, with the mainstay of treatment being a wide surgical resection to remove the whole tumour, but this can be challenging due to the infiltrative nature of MFSs through fascial planes. Appropriate pre-operative imaging is therefore essential for surgical planning. Currently, MRI imaging is the modality of choice to assess the soft-tissue extent of MFSs; however, it does not always reliably predict tumour extent, especially when an MRI shows high-signal curvilinear projections, known as “tails”, which often represent tumour extension and increase the risk of positive margins and local recurrence. **Methods**: This feasibility study therefore aimed to investigate whether the addition of an FDG PET-MRI and DWI MRI is superior for the local staging of MFSs compared to a standard MRI, and to assess its practicality for clinical use. **Results**: Of the eight patients recruited, six completed the required scans, proceeded to surgery, and were included in the data analyses. Five of the six patients had close (<2 mm) or positive margins requiring re-excision. **Conclusions**: Our results show that combining an FDG-PET and DWI MRI may offer a more accurate local staging of MFSs than a conventional MRI; however, a larger prospective trial is needed to further investigate this pilot data. Nevertheless, this novel feasibly study demonstrates the potential use of PET-MRI and DWI for improving pre-operative planning prior to the surgical resection of MFSs.

## 1. Introduction

Myxofibrosarcomas (MFSs) are aggressive soft-tissue sarcomas (STSs) that often arise in the extremities and are the most common subtype in elderly patients [1]. MFSs are characteristically invasive, frequently demonstrating significant infiltration along the fascial planes, often well beyond the extent of macroscopic disease, resulting in the need for large resection margins [2,3]. Unfortunately, an MFS is associated with higher positive margin rates (up to 43%) [4] and lower rates of local control, with local recurrence (LR) rates of over 30% [5,6], higher than that for other STS subtypes [2]. The risk of LR is significantly increased if the resection margins are inadequate [7], making them a particularly important and challenging subgroup of sarcomas for surgeons to manage. A wide surgical resection is the mainstay of treatment; however, a multidisciplinary approach at a regional sarcoma service is essential.

At present, surgeons base their resections on pre-operative magnetic resonance imaging (MRI), which is the current standard of care, along with visualisation and palpation of the tumour intra-operatively [8,9]. The standard MRI sequences employed at the time of this study to assess the local tumour extent of MFS include T1, spin echo T2, Short Tau Inversion Recovery (STIR), and Fat Saturation (Fat Sat) Proton Density (PD). MRI imaging can, however, lead to further diagnostic uncertainty with regards to the extent of a tumour. Curvilinear projections, known as ‘tails’, are defined as projections along the fascial plane with the same signal intensity as the principal mass, and typically have the same enhancement after a gadolinium-based contrast material injection [10], and are seen in up to 77% of MFSs [11]. These tails have previously been shown to represent an extension of microscopic disease along the fascial plane when MRI scans are correlated with the histopathological assessment [12]. The highly infiltrative nature of MFSs makes precise radiological mapping of the disease difficult. Surgically, this is important; the complete excision of a tumour is critical with regards to the oncological outcome [13,14,15], but the over-resection of normal tissues can undoubtedly lead to poorer physical function.

As such, there is a need for improved imaging modalities to guide surgeons in their resections. The largest advancement in recent years has been fluorescence-guided surgery (FGS); FGS with indocyanine green (ICG) has been shown to potentially reduce the positive margin rate in sarcoma surgeries [16].

An evolving area of pre-operative imaging is positron-emitting tomography (PET), typically using fluorodeoxyglucose (FDG), a radioactive form of glucose, as the tracer [17]. FDG accumulates in higher amounts in tissues that are more metabolically active, such as cancer. Most commonly, PET is fused with a computed tomography (CT) scan, resulting in a hybrid of cross-sectional imaging and metabolic activity [18]. The main use of this, at present, is during the staging process, to look for metastases; to characterise equivocal findings, such as indeterminate lymph nodes; and to assess the response to treatments [9,18]. PET images can also be fused with MRIs, allowing for improved soft-tissue assessment, including, for example, the differentiation of viable tumour and scar tissue [18]; the use of PET-MRI for the assessment of primary tumours has not yet been established, however. The use of FDG PET-MRI for breast cancer diagnosis is well described, and has shown improved specificity compared to a standard MRI alone [19], although it struggles to characterise lesions <10 mm in diameter [20]. Its ability to assess local tumour extent has also been assessed, although no additional disease has been identified compared to a standard MRI alone [21,22,23], although it may improve the detection of nodal disease [22,23]. PET-MRI is a developing area in the field of abdominal pelvic oncology [24], since MRIs are being increasingly used for the staging and restaging of oncological lesions of the abdomen and pelvis. PET-MRI provides superior soft-tissue characterisation and can differentiate between benign and malignant tissue activity better than PET/CT, where the physiological activity of highly metabolic tissues can mimic malignancy.

A diffusion-weighted MRI (DWI) has become increasingly used in the assessment of several malignancies, as it exploits the ability of an MRI to assess the restricted diffusion of water molecules in tissues, and has been shown to offer promise for differentiating between benign and malignant soft-tissue tumours, as well as the assessment of tumour margin infiltration in soft-tissue sarcomas [25,26].

To date, there have been few studies on the efficacy of PET-MRI imaging for the surgical planning of sarcomas. A previous study [27] compared the diagnostic accuracy of a PET-MRI to an MRI alone for the detection of local recurrence of STSs after an initial resection, showing a higher detection rate of tumour recurrence with PET-MRI. We hypothesise that a combined PET-MRI may offer added utility for the surgical planning of MFSs compared to the current standard MRI techniques.

## 2. Materials and Methods

**Patients:** This was a single-centre pilot study undertaken at the Northern Centre for Cancer Care, Newcastle upon Tyne Hospitals NHS Foundation Trust, Newcastle upon Tyne, United Kingdom. Patients with a histologically confirmed diagnosis of myxofibrosarcoma planned for definitive surgical management between August 2017 and December 2018 at the North of England Bone and Soft Tissue Tumour Service were considered for this feasibility study. Patients were excluded if not scheduled for curative resection. Ethical approval for this study was obtained from the North East Tyne & Wear Research Ethics Committee (REC Reference 16/NE/0324). Eligible patients were identified during bone and soft-tissue sarcoma multidisciplinary meetings and by out-patient clinics. All patients signed a formal informed consent form prior to inclusion.

**Imaging**: All participants underwent an additional PET-MRI scan, irrespective of previous standard-of-care imaging. Pre-operative standard-of-care MRI sequences (T1, spin echo T2, Short Tau Inversion Recovery (STIR), and Fat Saturation (Fat Sat) Proton Density (PD)) were performed, as well as additional diffusion-weighted imaging (DWI), with diffusion weightings of b50 and b800 and apparent diffusion coefficient (ADC) maps, alongside FDG PET. A dose of 3 MBq/Kg (300 MBq maximum) 18F-FDG was used. Participants were injected with FDG whilst lying on the scanning bed and MRI sequences of the primary tumour were acquired during the 60 min FDG uptake period. After a short break to allow for bladder emptying, whole-body PET-MRI images were acquired, which included skull base to mid-thigh (with extension to include the joint below the tumour when needed). The acquisition started as close as possible to 65 min post injection, using 4 min bed positions. MRI sequences used during this whole-body acquisition were coronal STIR in addition to the standard GE MR attenuation correction protocol.

For standard MRI and standard MRI + DWI acquisitions, tumour volumes and the presence or absence of a tail were assessed by a single consultant sarcoma radiologist with over 10 years’ experience. For standard MRI with FDG PET, tumour volume, SUV max, and the presence or absence of a tail was assessed by a radionuclide consultant radiologist with 6 years’ experience. When combining standard MRI, DWI, and FDG PET, images were reviewed jointly. For the purposes of this study, a tail was defined on imaging as a curvilinear projection with increased signal or activity extending from the primary tumour along a fascial plane. Following surgical resection, histopathological samples and pathology reports were reviewed to identify information about the margin status, area of highest Ki67 activity, and highest mitotic count in 10 high-power fields. Clinical records were assessed to determine whether re-excision was required. Tumour volume measurements were performed using the following: (1) standard MRI sequences, (2) standard MRI with diffusion-weighted imaging (DWI), (3) standard MRI with PET, and (4) standard MRI with both DWI/PET, and were compared for each participant. PET and MRI images were acquired simultaneously to allow for accurate comparison. In cases that resulted in a positive surgical margin, retrospective comparison between standard MRI and the experimental modalities was performed to identify any unique radiographic features on PET imaging. All imaging was performed on a GE Healthcare Signa PET-MRI scanner (GE Healthcare, Buckinghamshire, UK). Hermes Medical Solutions Software V6.1 (Stockholm, Sweden) was used to manually draw and calculate the tumour volume in each case. The operating surgeon was blinded to the investigational imaging for pre-operative workup unless metastases not previously known about were demonstrated.

**Statistical Analysis**: Paired Student’s *t*-tests were performed to determine differences in volume detected between imaging modalities, whilst Spearman’s rank correlation was conducted to determine the relationship between SUV max and both Ki67 and mitotic index. All analyses were performed on GraphPad Prism V.10.0.0 for macOS (GraphPad Software, Boston, MA, USA).

## 3. Results

**Figure 1:**
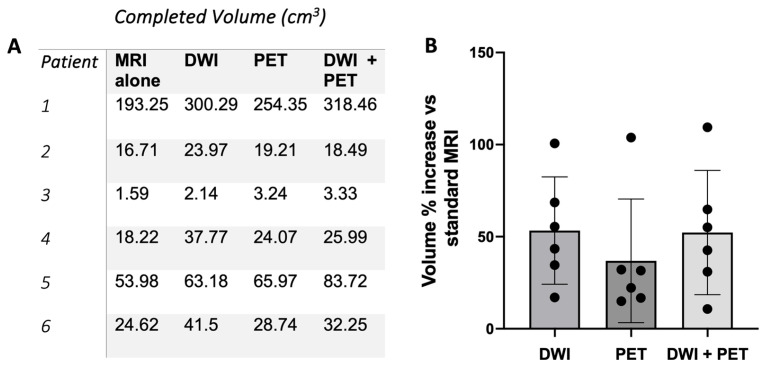
The difference in the calculated tumour volume across the four imaging modalities (**A**), and the percentage increase in tumour volume seen with DWI, PET, and DWI + PET compared to standard MRI alone (**B**).

Of the eight patients recruited, six had evaluable whole-body MRI scans across all the modalities, proceeded to surgery, and were included in the tumour volume analysis (one patient did not proceed to surgery and a complete PET-MRI dataset was not available for one patient). The total tumour volume was increased based on the assessment of the DWI, PET, and DWI + PET imaging compared to the standard MRI alone (Figure 1A), with a mean increase of 54.4%, 25.6%, and 51.8%, respectively, although these increases were not significant (*p* = 0.162, *p* = 0.184, *p* = 0.201). Despite using whole-body-sweep PET-MRI imaging for the six patients in this study, no metastatic disease was identified.

**Figure 2:**
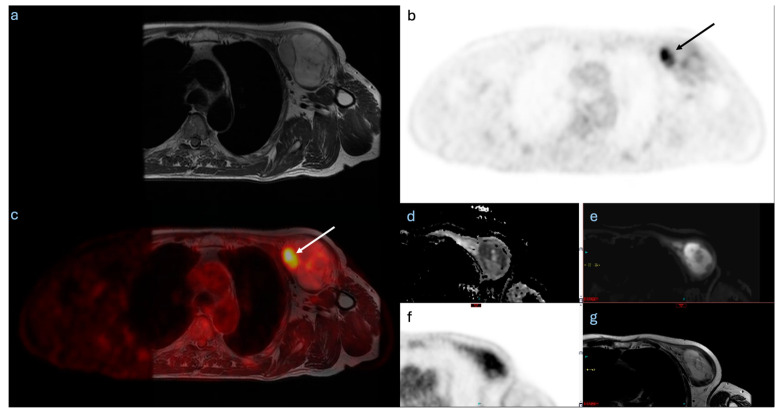
(**a**) Standard T1 MRI of grade 3 myxofibrosarcoma of the left chest wall, (**b**) FDG PET, and (**c**) fused PET-MRI images showing variable FDG uptake across the tumour. (**d**,**e**) Diffusion-weighted images (DWIs) and ADC. (**f**) FDG PET and (**g**) standard T1 MRI assessment of a myxofibrosarcoma tail. The black and white arrows for the FDG PET (**b**) and fused PET-MRI (**c**), respectively, identify the focus of higher FDG activity within the tumour, compatible with tumour heterogeneity and varied FDG uptake.

Of the six patients included, one patient had a positive surgical margin and underwent re-excision (Table 1). Notably, the PET and DWI imaging in this case identified FDG uptake and restricted diffusion with a tail that was more conspicuous on novel imaging compared to a standard MRI (Figure 2). Four additional patients had close margins (tumour cells within 2 mm of the resection margin) following the initial resection, and so underwent re-excision given the locally invasive nature and high risk of recurrence of the MFSs (Table 1) [28]. All five re-excisions had subsequent clear margins. One patient did not require a re-excision due to an adequate margin >2 mm on the index resection, and had the smallest increase in volume on the PET and DWI + PET compared to the standard MRI, of 15% and 10.7%, respectively. When analysing the tails specifically, four of the six tumours demonstrated tails on the MRI. These were present on the standard MRIs but were rated as more conspicuous on the DWIs for three of the four tumours. In one of these three cases, there was also restricted diffusion when compared to the ADC map, as well as FDG uptake on the PET. FDG uptake was not identified in the other three tails, but the tails were too thin for a complete PET characterisation.

**Figure 3:**
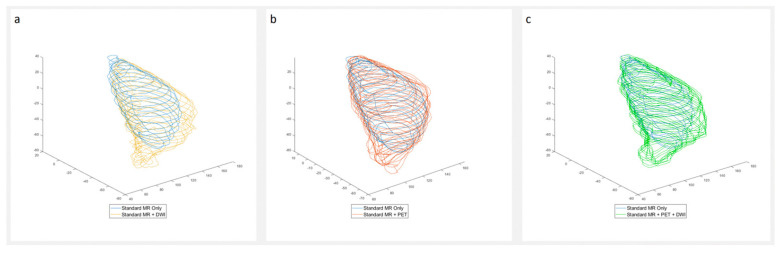
Images comparing the tumour volumes drawn with (**a**) standard MRI vs. standard MRI + DWI, (**b**) standard MRI vs. standard MRI + PET, and (**c**) standard MRI vs. standard MRI + PET + DWI in a patient with positive margins at surgery.

We assessed the tumour histology following surgical resection to compare the markers of tumour proliferation (Ki67 and mitotic index) with the overall tumour SUV max value obtained from the FDG PET-MRI imaging (Table 2 and Figure 3). In this small study, we observed a positive correlation between the Ki67 and SUV max, with a Spearman’s rank of rs = 0.820 and p (2 tailed) = 0.0458. The correlation between the mitotic index and SUV max was not significant, with rs = 0.6 and p (2 tailed) = 0.208. This may suggest that PET-MRI imaging could have a prognostic utility for predicting highly proliferative and aggressive myxofibrosarcomas, whilst also improving the visual assessment of tumour size, shape, and the presence of tails; however, a larger study would be required to prove this effect.

## 4. Discussion

In this feasibility study, both the DWI and PET increased the perceived tumour volume as determined by the expert radiologists in all six cases. The mean percentage increase for the combined DWI and PET-MRI vs. standard MRI was 51.8%. In the majority of tumours, this increase is likely to be a combination of a true increase in the appreciated tumour volume due to increased tumour conspicuity with the novel sequences, as well as secondarily due to the reduced spatial resolution of the PET and DWI acquisitions compared to some of the standard MRI sequences. The reduced resolution is likely to have a greater effect on the percentage change in volumes of small tumours, given the relatively fixed spatial resolution of the techniques for both small and large tumours. The increased conspicuity is highlighted by the expert radiologists’ observation that tumour was more easily seen in some cases using the novel sequences when compared to the standard MRI imaging. In fact, tumour extension, in addition to that seen on the standard MRI and not relating to the tail, was identified on the FDG PET in two of the six cases and on the DWI in one case. However, the limitations of this study include the subjectiveness surrounding radiologists’ interpretations of images from a small number of patients, and the fact that it was not possible to accurately correlate the tumour sizes with the histopathology due to the shrinkage and altered shape of the specimens post removal. Although it is strongly suggestive from these data that the increased perceived tumour volume on the PET-MRI represents the true tumour size, further work is required to confirm this.

Both the DWI and PET demonstrated disease at the tail for the only tumour with disease extending through the resection margin. This radiological finding was in the region of the positive margin, suggesting that a PET-MRI would likely have been beneficial for surgical planning in this case. DWI and PET are, however, still of limited use in thin tails, given that some are only a few millimetres wide and contain only a small number of tumour cells. This small size is likely to have impacted their ability to identify FDG uptake and restricted diffusion in the tails.

Contrast-enhanced MRI sequences can be used in the assessment of myxofibrosarcomas. The presence of an enhanced tail sign is associated with a worse local recurrence-free survival [12]. In addition, the presence of peritumoral contrast enhancement has been shown to have a high sensitivity and better specificity than peritumoural oedema for predicting high-grade tumours [29,30]. In the largest myxofibrosarcoma series to date assessing prognostic factors, the percentage volume of the enhancing tumour did not significantly correlate with local recurrence, but an infiltrative pattern “tail sign” did [31].

The positive correlation demonstrated between the SUV max and Ki67 is not unexpected, as rapidly dividing cancer cells utilise more energy. In tumour cells, anaerobic glycolysis is favoured as per the Warburg effect [32]. The inefficiency of this pathway results in increased glucose use and therefore FDG uptake, hence the correlation between the SUV max, which is determined by the degree of FDG uptake, and the proliferation marker Ki67. A single patient developed local recurrence at the follow-up. Of interest, this tumour demonstrated the highest Ki67, mitotic figure and SUV max. FDG PET-MRI full body sweep to include skull base to mid-thigh (with extension to include the joint below the tumour when needed) did not demonstrate any metastases in this study. This is likely due to the small sample size; however, all the patients also underwent pre-operative staging CT scans prior to the decision regarding curative resection and study recruitment.

In terms of the clinical practicality of PET-MRI for patients, acquisition was challenging in 50% of the initial eight patients recruited. This was mainly due to the complexity and long length of the scanning, with a combination of standard and novel MRI sequences during the uptake period followed by a whole-body PET-MRI sweep, as well as the novel nature of the PET-MRI scanner that led to some technical issues. Therefore, the extended scanning times for patients, which many find uncomfortable, and the increase in costs are important considerations for the future use of PET-MRI in MFSs. Separating the PET-MRI into two shorter scans (an MRI with additional novel sequences and a separate FDG PET-CT or PET-MRI sweep) may be helpful in addressing patient comfort. This may also address concerns regarding the availability and higher cost of a PET-MRI [33]. Standardising imaging protocols across scanners in the future may also be challenging given the different vendors and sequences available.

An important aspect of this study was to show the feasibility of PET-MRI in MFSs, in readiness for future technologies, including targeted PET tracers. Recent work on the development of a dual-modal PET/Near-Infrared Fluorescence (NIRF) radioimmunoconjugate has shown promise in a dedifferentiated sarcoma mouse model [34]. The combination of novel targeted tracers with pre-operative PET and MRI sequences will likely enhance the utility of this imaging modality for accurate pre-operative planning. It is also likely to improve the specificity of MFS tail detection, which is a current challenge for both standard MRI and PET-MRI and aids in the identification of metastatic disease [35,36]. A total-body PET-CT, where the body can be imaged in one field of view, has recently been developed. This has an improved resolution, signal-to-noise ratio, and lesion detection capability, which warrants consideration in the further investigation of MFS pre-operative planning [37]. This study provides novel data for the practicability and clinical use of PET-MRI in a sarcoma patient cohort, and future studies will aim to build on this work in a larger patient cohort. Building on this work, a PET-MRI as a tool for simultaneously assessing the novel biomarkers of different sarcoma subtypes is likely to be of great use in both animal and human studies.

## 5. Conclusions

Our results show that both FDG PET and DWI MRI are feasible for use in this population and may offer a more accurate local staging of MFSs due to increased tumour volume identification; however, a larger prospective trial is needed to further investigate this pilot data. Nevertheless, this novel feasibility study demonstrates the potential use of PET-MRI and DWI for improving the pre-operative planning for surgical resections of MFSs. This will be an important future consideration for helping to reduce the high positive margin rate of MFSs and improving patient outcomes.

## Figures and Tables

**Table 1:** Patient demographics and clinical outcomes of the six patients included in this feasibility study.

Patient	Gender	Age at Scan	Time from Scan to Surgery (Days)	Margin Status	Re-Excision	Local Recurrence
1	M	63	11	Positive	Y	N
2	M	65	2	Negative	N	N
3	M	67	3	Close	Y	N
4	M	54	1	Close	Y	N
5	M	77	8	Close	Y	Y
6	M	81	8	Close	Y	N

**Table 2:** Comparison of histological markers of proliferation and SUV max values of the tumours on FDG PET-MRI imaging.

Case	Ki67	Mitotic Rate	SUV Max
1	66	35	7
2	50	29	5.4
3	30	14	2.5
4	50	26	6
5	80	110	29.3
6	50	23	8.5

## Data Availability

The data are available upon reasonable request from the authors.

## Abbreviations

The following abbreviations are used in this manuscript:

CT	Computed Tomography
DWI	Diffusion-Weighted Imaging
FDG	[^1^⁸F] Fluorodeoxyglucose
ICG	Indocyanine Green
MFS	Myxofibrosarcoma
MRI	Magnetic Resonance Imaging
NIRF	Near-Infrared Fluorescence
PET	Positron Emission Tomography
STS	Soft-Tissue Sarcoma
SUV	Standardised Uptake Value
